# Relations of Lifestyle Behavior Clusters to Dyslipidemia in China: A Compositional Data Analysis

**DOI:** 10.3390/ijerph18157763

**Published:** 2021-07-22

**Authors:** Xiaona Na, Yangyang Chen, Xiaochuan Ma, Dongping Wang, Haojie Wang, Yang Song, Yumeng Hua, Peiyu Wang, Aiping Liu

**Affiliations:** 1 Department of Social Medicine and Health Education, School of Public Health, Peking University Health Science Center, Beijing 100191, China; naxiaona@bjmu.edu.cn (X.N.); 1911210136@bjmu.edu.cn (Y.C.); 1510306124@pku.edu.cn (X.M.); hyumengfa@163.com (Y.H.); wpeiyu@bjmu.edu.cn (P.W.); 2 Wuhai Center for Disease Control and Prevention, Inner Mongolia 016099, China; wdping3178@163.com (D.W.); jkwhj@163.com (H.W.); song2003yang@163.com (Y.S.)

**Keywords:** lifestyle behavior cluster, dyslipidemia, 24-h time-use, compositional data, dietary pattern

## Abstract

Dyslipidemia is associated with lifestyle behaviors, while several lifestyle behaviors exist collectively among some populaitons. This study aims to identify lifestyle behavior clusters and their relations to dyslipidemia. This cross-sectional study was conducted in Wuhai City, China. Cluster analysis combined with compositional data analysis was conducted, with 24-h time-use on daily activities and dietary patterns as input variables. Multiple logistic regression was conducted to compare dyslipidemia among clusters. A total of 4306 participants were included. A higher prevalence of newly diagnosed dyslipidemia was found among participants in cluster 1 (long sedentary behavior (SB) and the shortest sleep, high-salt and oil diet) /cluster 5 (the longest SB and short sleep), relative to the other clusters in both age groups (<50 years and ≥50 years). In conclusion, unhealthy lifestyle behaviors may exist together among some of the population, suggesting that these people are potential subjects of health education and behavior interventions. Future research should be conducted to investigate the relative significance of specific lifestyle behaviors in relation to dyslipidemia.

## 1. Introduction

Cardiovascular disease (CVD) is one of the most threatening diseases, accounting for 31% of mortality globally in 2016 [1]. Estimates of 422.74 million cases and 17.92 million deaths of CVD existed in 2015 around the world [2]. In China, especially, an estimated number of 93.8 million prevalent cases of CVD in 2016 was more than twice those in 1990 (40.6 million) [3]. The number of deaths owing to CVD increased from 2.51 million in 1990 to 3.97 million in 2016 [3].

Dyslipidemia refers to several lipid disorders that are characterized by at least one of following: raised total cholesterol (TC), raised triglycerides (TG), raised low-density lipoprotein cholesterol (LDL-C), and low high-density lipoprotein cholesterol (HDL-C) [4]. Dyslipidemia, a leading contributor to CVD, has become a global public health concern [5,6]. According to previous studies, the prevalence of dyslipidemia increased from 18.60% in 2007 to 36.46% in 2017 in China [7,8].

In addition, abnormal biomarkers of dyslipidemia could also lead to other chronic non-communicable diseases among some populations [9,10]. For example, a study showed increased atherosclerosis extension in prediabetes and newly diagnosed type 2 diabetes subjects with high TG/HDL compared to those with low TG/HDL [10].

In the past decades, with the rapid development of the economy, the living standard of Chinese people has been constantly improved. An increasing proportion of animal fat, oily, and salty foods are consumed in people’s diets, along with less physical activity (PA) and more sedentary behavior (SB) conducted among Chinese people. Regular lifestyle behaviors play significant roles in the prevention of dyslipidemia and other cardio-metabolic disorders [5]. Recent studies indicated that several lifestyle behaviors were associated with the risk of dyslipidemia [11,12,13]. They showed that proper PA, less SB, and healthy diet might decrease the occurrence and development of dyslipidemia. However, previous studies mainly focused on individual behavior separately [11]. It is crucial to investigate the comprehensive impact of these lifestyle behaviors instead of focusing on individual behaviors. Determining the cluster patterns of lifestyle behaviors will contribute to an in-depth understanding of people’s lifestyles impacting well-being. Recent researches have explored lifestyle behavior patterns and their associations with chronic non-communicable diseases among adults [14,15]. These studies identified several clusters that might be beneficial in the prevention of chronic non-communicable diseases by cluster analyses, and put forward relative policy recommendations.

People’s daily activities mainly comprise PA, SB, and sleep [16,17]. Time spent on one activity necessarily displaces that on others, that is, time spent on different activities is intrinsically co-dependent, finite, and subject to collinearity. Therefore, this kind of data is identified as compositional data [17,18]. It may be inappropriate to use traditional statistical analyses to process compositional data, since it may generate misleading results with some effects being misestimated or obscured [16]. To date, little about the combined effect of time-use on daily activities is known.

To address these gaps in the research literature, we aimed to identify lifestyle behaviors among participants in Wuhai City, Inner Mongolia, China, applying compositional data analysis and exploring the associations between these clusters and dyslipidemia.

## 2. Materials and Methods

### 2.1. Ethical Consideration

This study was approved by the Peking University Biomedical Ethics Committee (approval number IRB00001052-16022). The approval conformed to the provisions of the 1995 Declaration of Helsinki (revised in Edinburgh in 2000). All participants provided their written informed consent prior to the conduct of the survey.

### 2.2. Participants

This cross-sectional study was conducted based on the project of Adult Chronic Disease and Prevalence Factors Monitoring from June 2014 to October 2014 in Wuhai City. People who aged 18–79 years old and who lived locally for at least six months in the past year were eligible to participate in this study. Multiple stratified cluster random sampling was performed to guarantee the representativeness of sample, comprising two steps. First, 20 streets of Haibowan District, Hainan District, and Wuda District were selected by random sampling. Second, stratified sampling was performed according to participants’ employment status in each street. Employed participants were sampled from 105 work units according to local industry structure for males under 55 years and females under 50 years. Systematic sampling was conducted by employee number to determine the final sample. Unemployed participants were sampled from 100 households in 2 resident committees for males at least 55 years and females at least 50 years, and ultimate participants enrolled were selected by Kish in each household. Participants were excluded if they were pregnant or had neurocognitive difficulties.

### 2.3. Data Collection

Participants were required to complete a questionnaire, laboratory measurements, and a physical examination with the assistance of trained surveyors. The questionnaire was well designed and contained: (1) sociodemographic information, such as gender, age, education, and occupation; (2) information about personal lifestyle behaviors, including activities (PA, SB, and sleep), dietary intake, smoking status, and alcohol intake; and (3) information about history of chronic non-communicable diseases (dyslipidemia, hypertension, and diabetes).

Activities under investigation comprised PA, SB, and sleep. PA was measured with the long volume of International Physical Activity Questionnaire (IPAQ), an effective and widely used questionnaire for examining PA intensity, frequency, and cumulative time in adults [19,20,21]. Dietary intake was measured by a food frequency questionnaire (FFQ), which was proved to have moderate reliability and validity in Chinese adults [22,23,24]. FFQ comprised thirteen food categories relevant to local culture, and frequency (never, monthly, weekly, daily) and quantity consumed each time during the past month were investigated. Data on history of chronic non-communicable diseases were obtained from answers to the question “Have you ever been diagnosed as having dyslipidemia, diabetes, or hypertension by a doctor in a community hospital or above?”

Physical examination and laboratory measurements were performed to obtain data on physiological indexes. Fasting venous blood samples were collected with disposal vacuum blood collecting tubes. The concentrations of TC, TG, HDL-C, and LDL-C were measured by local second-class hospitals, where the equipment, instruments, and reagents conformed with the national production and using standard. Weight and height were measured by standard instruments, with participants wearing light clothing and no shoes.

### 2.4. Definition and Group

Lifestyle behavior clusters in this study were identified as the aggregation of 24-h time-use of activities (PA, SB, and sleep) and dietary patterns, which were the most important modified behaviors to improve dyslipidemia. PA intensity was classified according to IPAQ criteria [25], that is, PA was divided into 3 intensities, comprising vigorous intensity physical activity (VPA), moderate intensity physical activity (MPA), and light intensity physical activity (LPA), namely walking. Some researchers have shown that MPA and VPA might decrease risk of chronic non-communicable diseases, including dyslipidemia [11,26,27,28]. Therefore, MPA and VPA were merged to be identified as moderate-to-vigorous intensity PA (MVPA). Dietary pattern was identified according to principal component analysis, a detailed method of which was illustrated in Section 2.5.

Dyslipidemia was defined according to Chinese Guidelines for the Management of Dyslipidemia in Adults (2016 Revision) criteria, as the presence of one or more of the following factors: (1) serum TC concentration of 5.2 mmol/L or greater; (2) TG concentration of 1.7 mmol/L or greater; and (3) HDL-C concentration of under 1.0mmol/L [29]. Specifically, newly diagnosed dyslipidemia was defined as a participant who met the diagnostic criteria mentioned above and had not been diagnosed as dyslipidemia by a doctor in a community hospital or above. A prevalent case was defined as a participant who had been diagnosed with dyslipidemia by a doctor. BMI was calculated as the weight (kg) divided by the square of the height (m^2^), with a normal range of 18.5–23.9 kg/m^2^, considering the physiological characteristics of Chinese [30].

### 2.5. Statistical Analyses

**Figure 1:**
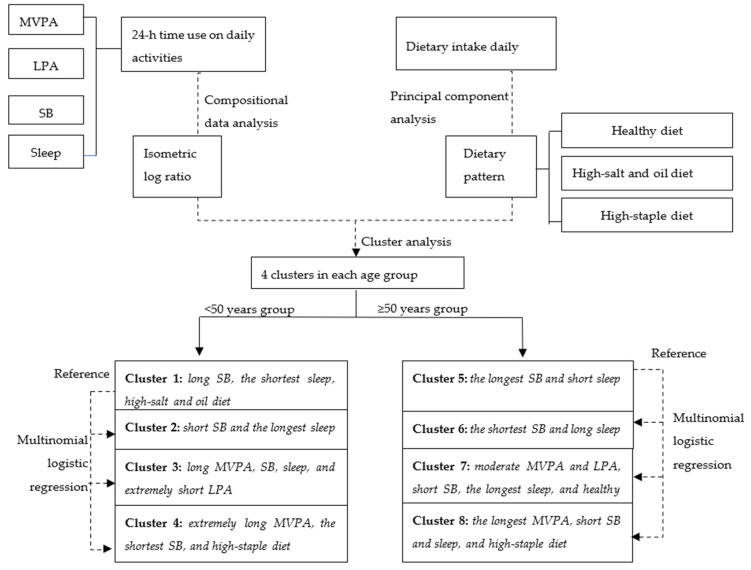
Flow chart of statistical analyses. SB, sedentary behavior; MVPA, moderate-to-vigorous intensity physical activity; LPA, light intensity of physical activity.

Flow chart of statistical analysis is presented in Figure 1. Data analyses consisted of: (1) describing sociodemographic characteristics by lifestyle behavior clusters of two age groups, (2) identifying the lifestyle behavior clusters of two age groups, and (3) exploring the relationships between clusters and dyslipidemia in two age groups.

Considering potential differences in lifestyle behaviors and risk of dyslipidemia across age groups, participants were divided into a <50 years group and a ≥50 years group [31,32,33,34,35]. In addition, in view of prevalence–incidence bias, newly diagnosed and prevalent cases were analyzed separately. Twenty-four-hour time-use on activities and dietary pattern were inputted to determine lifestyle behavior clusters. Twenty-four-hour time-use (compositional data) was transformed to isometric log ratios due to its closed nature, which contributed to solving the problem of collinearity in compositional data [36]. Compositional data means were used to describe the central tendency of twenty-four-hour time-use. Principal component analysis was conducted to identify three dietary patterns according to scree plot and professional interpretability, with daily intake of food as input variables: (1) healthy dietary pattern (positive loadings for vegetables, fruit, egg, dairy, soy, and its products), (2) high-salt and oil dietary pattern (positive loadings for grease, salt, and sauce), and (3) high-staple dietary pattern (positive loadings for cereal and tubers). Principal component scores representing the above dietary patterns were calculated for each participant and described with arithmetic means; an average score of at least 0.15 for a dietary pattern presented that a participant tended towards the corresponding pattern.

Scree plot was generated to determine the potential cluster number. Subsequently, a k-means partitioning cluster was used. An optimal number of 4 clusters was identified in two age groups based on the results of the scree plot and professional interpretability. To evaluate the stability of the cluster solution, a random subsample of each group (50% of participants in each age group) was clustered via conducting the same procedure. Agreements between solutions were relatively substantial (Cohen’s Kappa= 0.78 in <50 years group and 0.70 in ≥50 years group).

Dyslipidemia was compared across lifestyle behavior clusters in two age groups by multinomial logistic analysis with adjustment for covariates, containing age, gender, education, occupation, family annual income, BMI, smoke status, alcohol intake, and history of hypertension and diabetes.

Differences among clusters on sociodemographic characteristics and lifestyle behaviors were investigated by ANOVA (continuous variable) or *χ*^2^ tests (categorical variable). In this study, statistical significance was determined with *p* < 0.05 (two-tailed). Statistical analyses were performed with R 3.6.2 (R Development Core Team, Vienna, Austria).

## 3. Results

### 3.1. Sociodemographic Characteristics

Sociodemographic characteristics of participants are presented in Table 1. A total of 4306 participants were included, with an average age of 44.12 (standard deviation (SD) 12.91) years; participants in <50 years group and ≥50 years group averagely aged 36.95 (SD 8.20) and 58.96 (SD 6.60); 39.60% (1705/4306) and 60.40% (2601/4306) were men and women, respectively.

The difference of gender, education, occupation, BMI, smoking status, alcohol use status, and family annual income among clusters were statistically significant in the <50 years group. Age, gender, education, occupation, smoking status, alcohol use status, and family annual income among clusters had statistical differences in the ≥50 years group.

In the <50 years group, 965 (33.3%), 728 (25.2%), 527 (18.2%), and 674 (23.3%) belonged to cluster 1 to cluster 4, respectively, with cluster 1 accounting for the highest proportion. Compared with other clusters, cluster 1 was likely to be female, institution staff, non-smokers and non-drinkers, with a higher education level and a higher BMI ([26.27 ± 3.95] kg/m^2^), and a higher family annual income. In ≥50 years group, 309 (21.9%), 539 (38.2%), 196 (13.9%), and 368 (26.1%) belonged to cluster 5 to cluster 8, respectively, with cluster 6 accounting for the highest proportion. Compared with other clusters, cluster 6 was likely to be unemployed or retired, with a lower education level and family annual income.

### 3.2. Lifestyle Behavior Cluster Characteristics

Activity and diet characteristics of participants in two age groups are shown in Table 2, and descriptions of clusters are shown in Figure 1 according to the compared average activity time of each cluster with the corresponding mean time of overall population in two age groups. Four clusters were identified, and different cluster characteristics were observed in two age groups. Specifically, daily cumulative time of MVPA in the <50 years group and the ≥50 years group had little difference (187.32 min vs. 166.08 min), time of LPA in the <50 years group (111.05 min) was longer than that in the ≥50 years group (175.15 min), and time of SB (472.63 min vs. 297.94 min) and sleep (471.10 min vs. 438.04 min) in the <50 years group was almost longer than that in the ≥50 years group.

In particular, (1) Clusters 1/5 in both age groups were characterized by long cumulative time of SB and short cumulative time of sleep, but high-salt and oil diet existed in the <50 years group, and not in the ≥50 years group. (2) Lifestyle behavior characteristics of cluster 2/6 in both groups were similar, which were both characterized by short cumulative time of SB and long cumulative time of sleep, but cumulative time of sleep in the <50 years group was the longest and cumulative time of SB in the ≥50 years group was the shortest. (3) Lifestyle behavior characteristics in cluster 3 and cluster 7 were different. Cluster 3 was characterized by long cumulative time of MVPA, SB, sleep, and extremely short cumulative time of LPA; however, moderate cumulative time of MVPA and LPA, short cumulative time of SB, the longest cumulative time of sleep, and healthy diet in cluster 7. (4) Long cumulative time of MVPA, short cumulative time of SB, and high-staple diet were observed in clusters 4/8 in two age groups, but cumulative time of MVPA was extremely long and cumulative time of SB was the shortest in the <50 years group; and cumulative time of sleep was short in the ≥50 years group.

### 3.3. Cluster Memberships Relationship to Dyslipidemia

Blood lipid profiles among lifestyle behavior clusters are shown in Table 3. The results of MANOVA showed the lipid profiles were significantly different among clusters in both age groups. Especially, the difference of TC and LDL-C in the <50 years group, and TC and TG in the ≥50 years group, were statistically significant.

Among all the participants, 43.80% (1886/4306) were prevalent cases of dyslipidemia, 13.59% (585/4306) were newly diagnosed dyslipidemia cases, with the remaining 42.61% (1835/4306) not suffering from dyslipidemia. The associations between dyslipidemia and cluster memberships by multinomial logistic regression are shown in Table 4. A higher prevalence of newly diagnosed dyslipidemia was found among participants in cluster 1 relative to the other clusters (*p* < 0.01) in both age groups. No statistically difference of prevalent cases was found among clusters in both age groups. In particular, among people <50 years, participants in cluster 2 and cluster 4 had a significantly decreased prevalence of newly diagnosed dyslipidemia (OR_cluster2_ [odds ratio]: 0.688, 95% CI _cluster2_ [confidence interval]: 0.475–0.995; OR _cluster4_: 0.421, 95% CI _cluster4_: 0.277–0.640) relative to cluster 1 after covariate adjustment. Similarly, in the ≥50 years group, participants in cluster 2 and cluster 4 had a significantly decreased prevalence of newly diagnosed dyslipidemia (OR _cluster2_: 0.638, 95% CI _cluster2_: 0.412–0.988; OR _cluster4_: 0.365, 95% CI _cluster4_: 0.221–0.602) relative to cluster 1 after adjusting for potential confounders.

## 4. Discussion

This study aggregated compositional data of daily activities and dietary patterns into lifestyle behavior clusters and explored their relationships with dyslipidemia. Cluster analyses were conducted among participants aged < 50 years and ≥50 years because age might be a confounding factor influencing the association between lifestyle behavior and dyslipidemia. Cluster analysis is a statistical classification technique for discovering whether the individuals of a population fall into different groups, and the characteristics of the clusters were summarized by the results of cluster analysis. Time length of PA, SB, and sleep of a cluster was classified by comparing their time-use with the mean of PA, SB, and sleep in the total population in each age group.

Different lifestyle behavioral characteristics were found among clusters in two age groups. Besides, daily cumulative time of SB and sleep in the <50 years group was almost longer than that in the ≥50 years group. This phenomenon may result from greater need for sleep, prolonged sitting at work, pressure from every aspect, and relatively unhealthy lifestyle behavior among young people [34]. No obvious differences of MVPA and LPA existed between two age groups, except for extremely short cumulative time of LPA in the <50 years group, which may be due to more housework and physical exercises, instead of activities at work, conducted after retirement. This change has been found in previous research [37,38].

The average cumulative time of sleep in the <50 years group was higher than that in the ≥50 years group, but both groups met the criteria of the American Sleep Time Duration Recommendation [39]. The result of sleep variation by age was similar to a previous research [33]. However, short cumulative time of sleep and highest prevalence of newly diagnosed dyslipidemia in clusters 1/5 in both age groups indicated that a relationship between sleep and dyslipidemia might exist. Furthermore, several studies have shown that too long or too short sleep duration increased the risk of CVD, stroke, hypertension, diabetes, and metabolic syndrome [40,41,42]. Except for sleep duration, other aspects of sleep were proved to be related to chronic non-communicable diseases [43,44,45]. A study revealed the finding that sleep fragmentation was associated with higher blood glucose levels among African-Americans, and poor sleep efficiency and long wake after sleep onset increased risk of incident CVD [43]. As a result, comprehensive assessment of sleep should be conducted to explore its relationship with health outcomes.

Furthermore, results showed that part of the population focused on healthy lifestyle, but some unhealthy lifestyle behaviors might exist together, such as less PA, more SB, and unhealthy diet. This finding is in line with previous researches [11,46,47,48], suggesting these people are potential subjects of health education and behavior interventions in the future.

The results of multinomial logistic regression showed that risk of newly diagnosed dyslipidemia might be lower in cluster 2 and cluster 4, but not in cluster 3, compared with cluster 1 in both age groups. This finding indicated that less SB, combined with more MVPA, might decrease risk of newly diagnosed dyslipidemia effectively. However, conducting MVPA may have difficulties in relieving the harm of extremely long cumulative time of SB, so decreasing SB time may be relatively more effective than increasing MVPA time in decreasing risk of newly diagnosed dyslipidemia. However, no significant difference of prevalent dyslipidemia was observed among clusters in both age groups, which might be due to changes in lifestyle behaviors after suffering from dyslipidemia.

Age among clusters in ≥50 years varied significantly, with cluster 5 being the oldest. Therefore, age might be an influencing factor of selecting lifestyle behavior, and more SB and less PA might be conducted among participants with older age. The statistical difference on BMI among clusters in the <50 years group was significant, and cluster 5 had the highest BMI, which indicated that BMI might be associated with lifestyle behavior. Cluster 4 in the <50 years group showed different characteristics from the others. Extremely long cumulative time of MVPA, the shortest cumulative time of SB, and more staple foods intake were major characteristics in this cluster. Besides, cluster 4 tended to appear in participants who were male, less educated, consumed cigarettes and alcohol, and had low annual family income. This phenomenon may be explained by the “Health Worker Effect”, which means these young people who were comparatively better in physical fitness often choose manual work without paying much attention to a healthy lifestyle. Therefore, the association between extremely long cumulative time of MVPA and dyslipidemia is unclear. Previous research showed different findings for the health effects of MVPA [49,50], so further scientific research regarding this theme is needed.

The main strength of this study is the application of compositional data analysis to process 24-h time-use on activities, which are recommended for cluster analysis of compositional data. Furthermore, daily cumulative time of activities was considered to develop recommendation on daily PA, SB, and sleep. In addition, participants’ lifestyle behaviors were explored comprehensively, considering cumulative time of all intensities of PA, SB, sleep, as well as dietary patterns, which are the most crucial modified behavior factors to prevent dyslipidemia. To our knowledge, this study is the first to combine compositional data analysis with cluster analysis to identify lifestyle behavior clusters among adults, and their relationship with dyslipidemia. Previous studies have combined compositional data analysis and linear regression to examine the associations between 24-h time-use on activities and physiological indicators related to chronic non-communicable diseases without considering dietary pattern [17,51,52,53,54,55]. Recent studies have integrated compositional data analysis with cluster analysis to explore the relationships of adiposity or its indexes with 24-h time-use on activities among children and adolescents [16,56,57,58], but no such research has been conducted among adults.

Several limitations exist in our study. First, the cross-sectional design employed in this study impedes the inference of causation. Further prospective studies should be conducted. Second, information on activities and dietary intake was obtained by IPAQ and FFQ, so slight differences between self-reported data and the actual situation might exist. However, these questionnaires have been widely used among the Chinese population because of their substantial validity and reliability. Finally, the results cannot be generalized to other populations because of exploratory, data-driven nature of cluster analysis and principal component analysis.

## 5. Conclusions

In summary, this study adds to current evidence that less SB and more MVPA may decrease risk of dyslipidemia, and health education and behavior intervention should focus on the target population. Future studies should also be conducted to investigate the relative significance of specific lifestyle behaviors in relation to dyslipidemia, and make effective interventions.

## Figures and Tables

**Table 1:** Sociodemographic characteristics of different lifestyle behavior clusters.

	<50 Years Group						≥50 Years Group					
	Total	Cluster 1	Cluster 2	Cluster 3	Cluster 4	*p*	Total	Cluster 5	Cluster 6	Cluster 7	Cluster 8	*p*
	(*n* = 2894)	(*n* = 965)	(*n* = 728)	(*n* = 527)	(*n* = 674)		(*n* = 1412)	(*n* = 309)	(*n* = 539)	(*n* = 196)	(*n* = 368)	
**Age, years old, mean ± SD**	36.95 ± 8.20	37.62 ± 7.91	36.42 ± 8.74	37.08 ± 7.57	36.86 ± 8.35	0.353	58.96 ± 6.60	59.75 ± 7.06	58.16 ± 6.08	58.90 ± 6.07	57.86 ± 6.38	<0.01
**Gender, ** ***n*** **(%)**						<0.01						<0.01
Male	1096 (37.9)	330 (34.2)	267 (36.7)	194 (36.8)	305 (45.3)		609 (43.1)	162 (52.4)	234 (43.4)	81 (41.3)	132 (35.9)	
Female	1798 (62.1)	635 (65.8)	461 (63.3)	333 (63.2)	369 (54.7)		803 (56.9)	147 (47.6)	305 (56.6)	115 (58.7)	236 (64.1)	
**Education, ** ***n*** **(%)**						<0.01						<0.01
Junior high school or below	1376 (47.5)	275 (28.5)	407 (55.9)	155 (29.4)	539 (80.0)		1223 (86.6)	201 (65.0)	492 (91.3)	171 (87.2)	359 (97.6)	
Senior high school or above	1518(52.5)	690 (71.5)	321 (44.1)	372 (70.6)	135 (20.0)		189 (13.4)	108 (35.0)	47 (8.7)	25 (12.8)	9 (2.5)	
**Occupation, ** ***n*** **(%)**						<0.01						<0.01
Institution staff	1093 (37.8)	492 (51.0)	248 (34.1)	254 (48.2)	99 (14.7)		215 (15.2)	99 (32.0)	57 (10.6)	37 (18.9)	22 (6.0)	
Managers or technician	1625 (56.2)	441 (45.7)	404 (55.5)	252 (47.8)	528 (78.3)		422 (29.9)	81 (26.2)	136 (25.2)	56 (28.6)	149 (40.5)	
Unemployed or retired person	176 (6.1)	32 (3.32)	76 (10.4)	21 (4.0)	47 (7.0)		775 (54.9)	129 (41.7)	346 (64.2)	103 (52.6)	197 (53.5)	
**BMI (kg/m^2^), mean ± SD**	24.39 ± 3.76	26.27 ± 3.95	23.95 ± 3.25	24.95 ± 3.38	23.06 ± 3.58	<0.01	25.65 ± 3.81	26.05 ± 3.64	25.45 ± 3.57	25.63 ± 4.08	25.71 ± 3.47	0.38
**Smoking status, ** ***n*** **(%)**						<0.01						0.02
Yes	778 (26.9)	225 (23.3)	191 (26.2)	123 (23.3)	239 (35.5)		513 (36.3)	133 (43.0)	196 (36.4)	59 (30.1)	125 (34.0)	
No	2116 (73.1)	740 (76.7)	537 (73.8)	404 (76.7)	435 (64.5)		899 (63.7)	176 (57.0)	343 (63.6)	137 (69.9)	243 (66.0)	
**Alcohol use status, ** ***n*** **(%)**						<0.01						0.01
Yes	626 (21.6)	180 (18.7)	151 (20.7)	90 (17.1)	205 (30.4)		329 (23.3)	89 (28.8)	130 (24.1)	34 (17.3)	76 (20.7)	
No	2268 (78.4)	785 (81.3)	577 (79.3)	437 (82.9)	469 (69.6)		1083 (76.7)	220 (71.2)	409 (75.9)	162 (82.7)	292 (79.3)	
**Family annual income (yuan/year), ** ***n*** **(%)**						<0.01						<0.01
<30,000	1018 (35.2)	254 (26.3)	261 (35.9)	155 (29.4)	348 (51.6)		707 (37.9)	117 (37.9)	284 (52.7)	93 (47.4)	21 (57.9)	
30,000~79,999	1354 (46.8)	462 (47.9)	349 (47.9)	255 (48.4)	288 (42.7)		584 (41.4)	132 (42.7)	223 (41.4)	86 (43.9)	143 (38.9)	
80,000~	522 (18.0)	249 (25.8)	118 (16.2)	117 (22.2)	38 (5.6)		121 (8.6)	60 (19.4)	32 (5.9)	17 (8.7)	12 (3.26)	

In <50 years group: Cluster 1, long SB and the shortest sleep, high-salt and oil diet; Cluster 2, short SB and the longest sleep; Cluster 3, long MVPA, SB, sleep, and extremely short LPA; Cluster 4, extremely long MVPA, the shortest SB, and high-staple diet. In ≥50 years group: Cluster 5, the longest SB and short sleep; Cluster 6, the shortest SB and long sleep; Cluster 7, moderate MVPA and LPA, short SB, the longest sleep, and healthy diet; Cluster 8, the longest MVPA, short SB and sleep, and high-staple diet. Statistical differences of sociodemographic characteristics among clusters were compared by *χ*^2^ test. *p*, *p*-value; SB, sedentary behavior; MVPA, moderate-to-vigorous physical activity; LPA, light intensity of physical activity.

**Table 2:** Activities and dietary characteristics of lifestyle behavior clusters.

	<50 Years Group						≥50 Years Group					
	Total	Cluster 1	Cluster 2	Cluster 3	Cluster 4	*p*	Total	Cluster 5	Cluster 6	Cluster 7	Cluster 8	*p*
	(*n* = 2894)	(*n* = 965)	(*n* = 728)	(*n* = 527)	(*n* = 674)		(*n* = 1412)	(*n* = 309)	(*n* = 539)	(*n* = 196)	(*n* = 368)	
**Activities pattern, mean**						<0.01 *						<0.01 *
MVPA (min/day)	187.32	77.80 ^ab^	74.23 ^cd^	144.27 ^ace^	325.45 ^bde^	<0.01	166.08	99.48 ^abc^	76.55 ^ade^	130.06 ^bdh^	289.00 ^edh^	<0.01
LPA (min/day)	111.05	176.34 ^ab^	179.98 ^cd^	28.48 ^ace^	144.25 ^bde^	<0.01	175.15	147.27^abc^	180.00 ^ad^	178.31 ^be^	166.29 ^cde^	<0.01
SB (min/day)	472.63	544.32 ^abc^	250.53 ^ad^	695.64 ^bde^	246.85 ^ce^	<0.01	297.94	565.43 ^abc^	191.49 ^ade^	222.06 ^bd^	224.57 ^ce^	<0.01
Sleep (min/day)	471.10	451.60 ^abc^	493.27 ^ade^	481.20 ^bdh^	467.40 ^ceh^	<0.01	438.04	428.16 ^ab^	447.44 ^ac^	455.66 ^bd^	425.46 ^cd^	0.02
**Dietary pattern (principal component scores), mean**						<0.01 *						<0.01 *
Healthy diet	−0.06	0.03 ^a^	−0.12 ^ab^	−0.02	0.09 ^b^	<0.01	0.34	−0.06 ^abc^	−0.40 ^ade^	1.66 ^bdh^	−0.25 ^edh^	0.02
High-salt and oil diet	−0.04	0.18 ^ab^	−0.12 ^ac^	−0.10 ^d^	0.06 ^bcd^	<0.01	0.01	0.12 ^a^	−0.05 ^b^	−0.17 ^abc^	0.07 ^c^	0.04
High-staple diet	0.07	−0.25 ^abc^	0.04 ^ad^	−0.08 ^be^	0.39 ^cde^	<0.01	0.17	−0.01 ^a^	−0.18	0.24 ^ab^	0.15 ^c^	<0.01

In <50 years group: Cluster 1, long SB and the shortest sleep, high-salt and oil diet; Cluster 2, short SB and the longest sleep; Cluster 3, long MVPA, SB, sleep, and extremely short LPA; Cluster 4, extremely long MVPA, the shortest SB, and high-staple diet. In ≥50 years group: Cluster 5, the longest SB and short sleep; Cluster 6, the shortest SB and long sleep; Cluster 7, moderate MVPA and LPA, short SB, the longest sleep, and healthy diet; Cluster 8, the longest MVPA, short SB and sleep, and high-staple diet. * *p*-value was obtained by MANOVA. Superscript letters denote statistically significant pairwise comparisons (following Bonferroni correction of *p* < 0.008). *p*, *p*-value; SB, sedentary behavior; MVPA, moderate-to-vigorous intensity physical activity; LPA, light intensity physical activity; MANOVA, multivariate analysis of variance.

**Table 3:** Blood lipid profiles of lifestyle behavior clusters.

<50 Years Group							≥50 Years Group					
	Total (*n* = 2894)	Cluster 1 (*n* = 965)	Cluster 2 (*n* = 728)	Cluster 3 (*n* = 527)	Cluster 4 (*n* = 674)	*p*	Total (*n* = 1412)	Cluster 1 (*n* = 309)	Cluster 2 (*n* = 539)	Cluster 3 (*n* = 196)	Cluster 4 (*n* = 368)	*p*
						<0.01 *						<0.01 *
TC	4.76	4.83 ^a^	4.71	4.63 ^ab^	4.81 ^b^	<0.01	5.22	5.30 ^ab^	5.27	5.13 ^a^	5.14 ^b^	0.03
TG	1.62	1.63	1.62	1.55	1.64	0.30	1.96	2.27 ^abc^	1.88 ^a^	1.90 ^b^	1.84^c^	<0.01
HDL-C	1.30	1.28	1.31	1.31	1.31	0.98	1.33	1.32	1.32	1.33	1.34	0.25
LDL-C	2.86	2.98 ^abc^	2.83 ^a^	2.73 ^bd^	2.86 ^bcd^	<0.01	3.10	3.12	3.16	3.02	3.04	0.09

In <50 years group: Cluster 1, long SB and the shortest sleep, high-salt and oil diet; Cluster 2, short SB and the longest sleep; Cluster 3, long MVPA, SB, sleep, and extremely short LPA; Cluster 4, extremely long MVPA, the shortest SB, and high-staple diet. In ≥50 years group: Cluster 5, the longest SB and short sleep; Cluster 6, the shortest SB and long sleep; Cluster 7, moderate MVPA and LPA, short SB, the longest sleep, and healthy diet; Cluster 8, the longest MVPA, short SB and sleep, and high-staple diet. * *p*-value was obtained by MANOVA. Superscript letters denote statistically significant pairwise comparisons (following Bonferroni correction of *p* < 0.008). *p*, *p*-value; TC, total cholesterol; TG, triglyceride; HDL-C, high-density lipoprotein cholesterol; LDL-C, low-density lipoprotein cholesterol; SB, sedentary behavior; MVPA, moderate-to-vigorous intensity physical activity; LPA, light intensity physical activity; MANOVA, multivariate analysis of variance.

**Table 4:** Associations between dyslipidemia and lifestyle clusters among participants.

Newly Diagnosed Dyslipidemia				Prevalent Dyslipidemia			
Cluster	*n* (%)	Model 1 AOR (95% CI)	Model 2 AOR (95% CI)	Cluster	*n* (%)	Model 1 AOR (95% CI)	Model 2 AOR (95% CI)
**<50 years old**							
1	115 (11.9%) ^a^	1.000 (Reference)	1.000 (Reference)	1	385 (39.9%) ^a^	1.000 (Reference)	1.000 (Reference)
2	69 (9.5%)	0.654 (0.458, 0.936) *	0.688 (0.475, 0.995) *	2	298 (40.9%)	0.801 (0.560, 1.145)	0.875 (0.700, 1.095)
3	57 (10.8%)	0.738 (0.507, 1.074)	0.901 (0.604, 1.345)	3	187 (35.5%) ^b^	0.663 (0.368, 1.195)	0.824 (0.640, 1.059)
4	46 (6.8%) ^a^	0.355 (0.238, 0.529) ^†^	0.421 (0.277, 0.640) *	4	317 (47.0%) ^ab^	0.794 (0.557, 1.132)	0.848 (0.671, 1.073)
**≥50 years old**							
5	91(29.4%) ^a^	1.000 (Reference)	1.000 (Reference)	5	143 (46.3%) ^abc^	1.000 (Reference)	1.000 (Reference)
6	116(21.5%)	0.595 (0.400, 0.887) ^†^	0.638 (0.412, 0.988) *	6	265 (49.2%) ^a^	0.808 (0.559, 1.169)	0.824 (0.569, 1.194)
7	42(21.4%)	0.681 (0.406, 1.140)	0.800 (0.460, 1.392)	7	104 (53.1%) ^b^	1.050 (0.664, 1.659)	1.080 (0.680, 1.705)
8	49(13.3%) ^a^	0.300 (0.190, 0.470) ^†^	0.365 (0.221, 0.602) ^†^	8	187 (50.8%) ^c^	0.683 (0.462, 1.008)	0.702 (0.475, 1.039)

In <50 years group: Cluster 1, long SB and the shortest sleep, high-salt and oil diet; Cluster 2, short SB and the longest sleep; Cluster 3, long MVPA, SB, sleep, and extremely short LPA; Cluster 4, extremely long MVPA, the shortest SB, and high-staple diet. In ≥50 years group: Cluster 5, the longest SB and short sleep; Cluster 6, the shortest SB and long sleep; Cluster 7, moderate MVPA and LPA, short SB, the longest sleep, and healthy diet; Cluster 8, the longest MVPA, short SB and sleep, and high-staple diet. Model 1 was adjusted for age, gender, education, occupation, and family annual income. Model 2 was adjusted for BMI, smoke, alcohol, history of hypertension and diabetes based on model 1. * *p* < 0.05; ^†^ *p* < 0.01. Superscript letters denote statistically significant pairwise comparisons (following Bonferroni correction of *p* < 0.008). AOR, adjusted odds ratio; CI, confidence interval; SB, sedentary behavior; MVPA, moderate-to-vigorous intensity physical activity; LPA, light intensity physical activity.

## Data Availability

Data sharing is not applicable to this article.

## References

[1] World Health Organization Cardiovascular Diseases (CVDs). https://www.who.int/en/news-room/fact-sheets/detail/cardiovascular-diseases-(cvds).

[2] Roth G.A., Johnson C., Abajobir A., Abd-Allah F., Abera S.F., Abyu G., Ahmed M., Aksut B., Alam T., Alam K. (2017). Global, egional, and National Burden of Cardiovascular Diseases for 10 Causes, 1990 to 2015. J. Am. Coll. Cardiol..

[3] Liu S., Li Y., Zeng X., Wang H., Yin P., Wang L., Liu Y., Liu J., Qi J., Ran S. (2019). Burden of Cardiovascular Diseases in China, 1990–2016: Findings From the 2016 Global Burden of Disease Study. JAMA Cardiol..

[4] Musunuru K. (2010). Atherogenic Dyslipidemia: Cardiovascular Risk and Dietary Intervention. Lipids.

[5] Kopin L., Lowenstein C. (2017). Dyslipidemia. Ann. Intern. Med..

[6] Murphy A., Faria-Neto J.R., Al-Rasadi K., Blom D., Catapano A., Cuevas A., Lopez-Jimenez F., Perel P., Santos R., Sniderman A. (2017). World Heart Federation Cholesterol Roadmap. Glob. Heart.

[7] Zhao W.H., Zhang J., Zhai Y., You Y., Man Q.Q., Wang C.R., Li H., Li Y., Yang X.G. (2007). Blood lipid profile and prevalence of dyslipidemia in Chinese adults. Biomed. Environ. Sci..

[8] Tian Z., Li Y., Mao Z., Yu S., Wang Y., Liu X., Tu R., Zhang H., Qian X., Zhang X. (2018). Sex-specific relationship between visceral fat index and dyslipidemia in Chinese rural adults: The Henan Rural Cohort Study. Prev. Med..

[9] Scicali R., Di Pino A., Platania R., Purrazzo G., Ferrara V., Giannone A., Urbano F., Filippello A., Rapisarda V., Farruggia E. (2018). Detecting familial hypercholesterolemia by serum lipid profile screening in a hospital setting: Clinical, genetic and atherosclerotic burden profile. Nutr. Metab. Cardiovasc. Dis..

[10] Scicali R., Giral P., D’Erasmo L., Cluzel P., Redheuil A., Di Pino A., Rabuazzo A.M., Piro S., Arca M., Béliard S. (2021). High TG to HDL ratio plays a significant role on atherosclerosis extension in prediabetes and newly diagnosed type 2 diabetes subjects. Diabetes Metab. Res. Rev..

[11] Zhou J., Zhou Q., Wang D.P., Zhang T., Wang H.J., Song Y., He H.Z., Wang M., Wang P.Y., Liu A.P. (2017). Associations of sedentary behavior and physical activity with dyslipidemia. Beijing Da Xue Xue Bao Yi Xue Ban.

[12] Patnode C.D., Evans C.V., Senger C.A., Redmond N., Lin J.S. (2017). Behavioral Counseling to Promote a Healthful Diet and Physical Activity for Cardiovascular Disease Prevention in Adults Without Known Cardiovascular Disease Risk Factors: Updated Evidence Report and Systematic Review for the US Preventive Services Task Force. JAMA.

[13] Clifton P.M. (2019). Diet, exercise and weight loss and dyslipidaemia. Pathology.

[14] Villegas R., Yang G., Gao Y.T., Cai H., Li H., Zheng W., Shu X.O. (2010). Dietary patterns are associated with lower incidence of type 2 diabetes in middle-aged women: The Shanghai Women’s Health Study. Int. J. Epidemiol..

[15] Charreire H., Casey R., Salze P., Kesse-Guyot E., Simon C., Chaix B., Banos A., Badariotti D., Touvier M., Weber C. (2010). Leisure-time physical activity and sedentary behavior clusters and their associations with overweight in middle-aged French adults. Int. J. Obes..

[16] Pedišić Ž. (2014). Measurement issues and poor adjustments for physical activity and sleep undermine sedentary behaviour research--the focus should shift to the balance between sleep, sedentary behaviour, standing and activity. Kinesiology.

[17] Chastin S.F., Palarea-Albaladejo J., Dontje M.L., Skelton D.A. (2015). Combined Effects of Time Spent in Physical Activity, Sedentary Behaviors and Sleep on Obesity and Cardio-Metabolic Health Markers: A Novel Compositional Data Analysis Approach. PLoS ONE.

[18] Aitchison J. (1986). The Statistical Analysis of Compositional Data.

[19] Deng H.B., Macfarlane D.J., Thomas G.N., Lao X.Q., Jiang C.Q., Cheng K.K., Lam T.H. (2008). Reliability and validity of the IPAQ-Chinese: The Guangzhou Biobank Cohort study. Med. Sci. Sports Exerc..

[20] Fan M., Lyu J., He P. (2014). Chinese guidelines for data processing and analysis concerning the International Physical Activity Questionnaire. Chin. J. Epidemiol..

[21] Craig C.L., Marshall A.L., Sjöström M., Bauman A.E., Booth M.L., Ainsworth B.E., Pratt M., Ekelund U., Yngve A., Sallis J.F. (2003). International physical activity questionnaire: 12-country reliability and validity. Med. Sci. Sports Exerc..

[22] Ye Q., Hong X., Wang Z., Yang H., Chen X., Zhou H., Wang C., Lai Y., Sun L., Xu F. (2016). Reproducibility and validity of an FFQ developed for adults in Nanjing, China. Br. J. Nutr..

[23] Xue Y., Yang K., Wang B., Liu C., Mao Z., Yu S., Li X., Wang Y., Sun H., Wang C. (2020). Reproducibility and validity of an FFQ in the Henan Rural Cohort Study. Public Health Nutr..

[24] Hong X., Ye Q., Wang Z., Yang H., Chen X., Zhou H., Wang C., Chu W., Lai Y., Sun L. (2016). Reproducibility and validity of dietary patterns identified using factor analysis among Chinese populations. Br. J. Nutr..

[25] International Physical Activity Questionnaire: Long Last 7 Days Self-Administered Format. http://www.sdp.univ.fvg.it/sites/default/files/IPAQ_English_self-admin_long.pdf.

[26] Monda K.L., Ballantyne C.M., North K.E. (2009). Longitudinal impact of physical activity on lipid profiles in middle-aged adults: The Atherosclerosis Risk in Communities Study. J. Lipid Res..

[27] Liu Q., Liu F.C., Huang K.Y., Li J.X., Yang X.L., Wang X.Y., Chen J.C., Liu X.Q., Cao J., Shen C. (2020). Beneficial effects of moderate to vigorous physical activity on cardiovascular disease among Chinese adults. J. Geriatr. Cardiol..

[28] Beyer K.M.M., Szabo A., Hoormann K., Stolley M. (2018). Time spent outdoors, activity levels, and chronic disease among American adults. J. Behav. Med..

[29] (2018). 2016 Chinese guidelines for the management of dyslipidemia in adults. J. Geriatr. Cardiol..

[30] Chen C., Lu F.C. (2004). The guidelines for prevention and control of overweight and obesity in Chinese adults. Biomed. Environ. Sci..

[31] Feng Q., Yeung W.J., Wang Z., Zeng Y. (2019). Age of Retirement and Human Capital in an Aging China, 2015–2050. Eur. J. Popul..

[32] Liu H.H., Li J.J. (2015). Aging and dyslipidemia: A review of potential mechanisms. Ageing Res. Rev..

[33] Dorffner G., Vitr M., Anderer P. (2015). The effects of aging on sleep architecture in healthy subjects. Adv. Exp. Med. Biol..

[34] Nooyens A.C.J., Visscher T.L.S., Schuit A.J., van Rossum C.T.M., Verschuren W.M.M., van Mechelen W., Seidell J.C. (2005). Effects of retirement on lifestyle in relation to changes in weight and waist circumference in Dutch men: A prospective study. Public Health Nutr..

[35] Zhou Y., Wu J., Zhang S., Yan S., He L., Mkandawire N., Song X., Gan Y., Li W., Yang T. (2018). Prevalence and risk factors of physical inactivity among middle-aged and older Chinese in Shenzhen: A cross-sectional study. BMJ Open.

[36] Palarea-Albaladejo J., Martín-Fernández J.A., Soto J.A. (2012). Dealing with Distances and Transformations for Fuzzy C-Means Clustering of Compositional Data. J. Classif..

[37] Szinovacz M.E. (2000). Changes in housework after retirement: A panel analysis. J. Marriage Fam..

[38] Barnett I., van Sluijs E.M., Ogilvie D. (2012). Physical activity and transitioning to retirement: A systematic review. Am. J. Prev. Med..

[39] Hirshkowitz M., Whiton K., Albert S.M., Alessi C., Bruni O., DonCarlos L., Hazen N., Herman J., Katz E.S., Kheirandish-Gozal L. (2015). National Sleep Foundation’s sleep time duration recommendations: Methodology and results summary. Sleep Health.

[40] Smiley A., King D., Bidulescu A. (2019). The Association between Sleep Duration and Metabolic Syndrome: The NHANES 2013/2014. Nutrients.

[41] Krittanawong C., Kumar A., Wang Z., Jneid H., Baber U., Mehran R., Tang W.H.W., Bhatt D.L. (2020). Sleep Duration and Cardiovascular Health in a Representative Community Population (from NHANES, 2005 to 2016). Am. J. Cardiol..

[42] Wang Y.-H., Wang J., Chen S.-H., Li J.-Q., Lu Q.-D., Vitiello M.V., Wang F., Tang X.-D., Shi J., Lu L. (2020). Association of Longitudinal Patterns of Habitual Sleep Duration With Risk of Cardiovascular Events and All-Cause Mortality. JAMA Netw. Open.

[43] Yan B., Yang J., Zhao B., Fan Y., Wang W., Ma X. (2021). Objective Sleep Efficiency Predicts Cardiovascular Disease in a Community Population: The Sleep Heart Health Study. J. Am. Heart Assoc..

[44] Yano Y., Gao Y., Johnson D.A., Carnethon M., Correa A., Mittleman M.A., Sims M., Mostofsky E., Wilson J.G., Redline S. (2020). Sleep Characteristics and Measures of Glucose Metabolism in Blacks: The Jackson Heart Study. J. Am. Heart Assoc..

[45] Huang T., Mariani S., Redline S. (2020). Sleep Irregularity and Risk of Cardiovascular Events. J. Am. Coll. Cardiol..

[46] Boucher A.B., Adesanya E.A.O., Owei I., Gilles A.K., Ebenibo S., Wan J., Edeoga C., Dagogo-Jack S. (2015). Dietary habits and leisure-time physical activity in relation to adiposity, dyslipidemia, and incident dysglycemia in the pathobiology of prediabetes in a biracial cohort study. Metabolism.

[47] Qi L., Ding X., Tang W., Li Q., Mao D., Wang Y. (2015). Prevalence and Risk Factors Associated with Dyslipidemia in Chongqing, China. Int. J. Environ. Res. Public Health.

[48] Lira F.S., Rosa Neto J.C., Antunes B.M., Fernandes R.A. (2014). The relationship between inflammation, dyslipidemia and physical exercise: From the epidemiological to molecular approach. Curr. Diabetes Rev..

[49] Wicker P., Frick B. (2017). Intensity of physical activity and subjective well-being: An empirical analysis of the WHO recommendations. J. Public Health.

[50] Aune D., Norat T., Leitzmann M., Tonstad S., Vatten L.J. (2015). Physical activity and the risk of type 2 diabetes: A systematic review and dose-response meta-analysis. Eur. J. Epidemiol..

[51] Janurek J., Abdel Hadi S., Mojzisch A., Häusser J.A. (2018). The Association of the 24 Hour Distribution of Time Spent in Physical Activity, Work, and Sleep with Emotional Exhaustion. Int. J. Environ. Res. Public Health.

[52] McGregor D.E., Palarea-Albaladejo J., Dall P.M., Stamatakis E., Chastin S.F.M. (2019). Differences in physical activity time-use composition associated with cardiometabolic risks. Prev. Med. Rep..

[53] Rasmussen C.L., Palarea-Albaladejo J., Bauman A., Gupta N., Nabe-Nielsen K., Jørgensen M.B., Holtermann A. (2018). Does Physically Demanding Work Hinder a Physically Active Lifestyle in Low Socioeconomic Workers? A Compositional Data Analysis Based on Accelerometer Data. Int. J. Environ. Res. Public Health.

[54] Pelclová J., Štefelová N., Hodonská J., Dygrýn J., Gába A., Zając-Gawlak I. (2018). Reallocating Time from Sedentary Behavior to Light and Moderate-to-Vigorous Physical Activity: What Has a Stronger Association with Adiposity in Older Adult Women?. Int. J. Environ. Res. Public Health.

[55] Dumuid D., Lewis L.K., Olds T.S., Maher C., Bondarenko C., Norton L. (2018). Relationships between older adults’ use of time and cardio-respiratory fitness, obesity and cardio-metabolic risk: A compositional isotemporal substitution analysis. Maturitas.

[56] Dumuid D., Olds T., Lewis L.K., Martin-Fernández J.A., Barreira T., Broyles S., Chaput J.P., Fogelholm M., Hu G., Kuriyan R. (2018). The adiposity of children is associated with their lifestyle behaviours: A cluster analysis of school-aged children from 12 nations. Pediatr. Obes..

[57] Dumuid D., Olds T., Lewis L.K., Martin-Fernández J.A., Katzmarzyk P.T., Barreira T., Broyles S.T., Chaput J.P., Fogelholm M., Hu G. (2017). Health-Related Quality of Life and Lifestyle Behavior Clusters in School-Aged Children from 12 Countries. J. Pediatr..

[58] Wong M., Olds T., Gold L., Lycett K., Dumuid D., Muller J., Mensah F.K., Burgner D., Carlin J.B., Edwards B. (2017). Time-Use Patterns and Health-Related Quality of Life in Adolescents. Pediatrics.

